# Efficient production of 1,3-propanediol from crude glycerol by repeated fed-batch fermentation strategy of a lactate and 2,3-butanediol deficient mutant of *Klebsiella pneumoniae*

**DOI:** 10.1186/s12934-018-0921-z

**Published:** 2018-06-15

**Authors:** Baek-Rock Oh, Sung-Mok Lee, Sun-Yeon Heo, Jeong-Woo Seo, Chul Ho Kim

**Affiliations:** 1 Microbial Biotechnology Research Center, Jeonbuk Branch Institute, Korea Research Institute of Bioscience and Biotechnology (KRIBB), Jeonbuk, Jeongeup, 580-185 Republic of Korea; 2 Korea Institute of Ocean Science and Technology, Busan, 49111 Republic of Korea

**Keywords:** *Klebsiella pneumoniae*, Crude glycerol, 1,3-Propanediol, Repeated fed-batch fermentation, Mutant

## Abstract

**Background:**

1,3-Propanediol (1,3-PDO) is important building blocks for the bio-based chemical industry, *Klebsiella pneumoniae* can be an attractive candidate for their production. However, 1,3-PDO production is high but productivity is generally low by *K. pneumoniae*. In this study, repeated fed-batch cultivation by a lactate and 2,3-butanediol (2,3-BDO) deficient mutant of *K. pneumoniae* were investigated for efficient 1,3-PDO production from industrial by-products such as crude glycerol.

**Results:**

First, optimal conditions for repeated fed-batch fermentation of a Δ*ldhA* mutant defective for lactate formation due to deletion of the lactate dehydrogenase gene (*ldhA*) were determined. Maximal 1,3-PDO production level and productivity obtained by repeated fed-batch fermentation under optimized conditions were 81.1 g/L and 3.38 g/L/h, respectively, and these values were successfully maintained for five cycles of fermentation without any loss of fermentation capacity. This results were much higher than that of the normal fed-batch fermentation. The levels of 2,3-BDO, which is a major by-product, reaching up to ~ 50% of the level of 1,3-PDO, were reduced using a mutant strain [Δ(*ldhA als*)] containing an additional mutation in the biosynthetic pathway of 2,3-BDO (deletion of the acetolactate synthase gene). The levels of 2,3-BDO were reduced to about 20% of 1,3-PDO levels by repeated fed-batch fermentation of Δ(*ldhA als*), although maximal 1,3-PDO production and productivity also decreased owing to a defect in the growth of the 2,3-BDO-defective mutant strain.

**Conclusion:**

This repeated fed-batch fermentation may be useful for reducing the cost of 1,3-PDO production and may be promising industrialization prospect for the 1,3-PDO production.

## Background

1,3-PDO is a valuable chemical used principally in the synthesis of polymethylene terephthalates, wherein it is polymerized with terephthalates [10, 26]. Applications of these polymers in the manufacture of materials such as textile fibers, films, and plastics are increasing rapidly [11, 25]. 1,3-PDO building block is currently produced by chemical processes, such as hydroformylation of ethylene oxide or hydration of acrolein. However, the original route to 1,3-PDO production involved microbial fermentation using glycerol as a substrate [4, 7, 9, 18, 20]. Since, glycerol is a major by-product of the biodiesel industry, such biological conversion is currently receiving considerable attention.

**Fig. 1:**
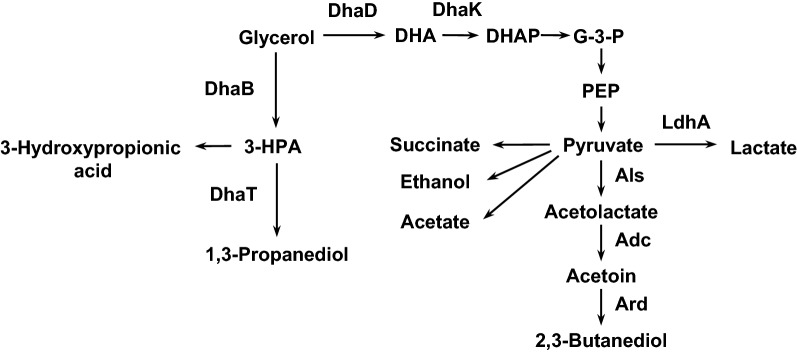
Glycerol metabolic pathway in *K. pneumoniae*. *DHA* dehydroxyacetone, *DHAP* dehydroxyacetone phosphate, *G-3-P* glycerol-3 phosphate, *PEP* phosphoenol pyruvate, *3-HPA* 3-hydroxypropion aldehyde, *DhaD* glycerol dehydrogenase, *DhaK* DHA kinase, *DhaB* glycerol dehydratase, *DhaT* 1,3-propanediol oxidoreductase, *LdhA* lactate dehydrogenase, *Als* acetolactate synthase, *Adc* acetolactate decarboxylase, *Ard* acetoin reductase

*Klebsiella pneumoniae* is typical of microorganism that produce 1,3-PDO from glycerol, and the relevant metabolic pathway has been well studied (Fig. 1) [11, 28]. Glycerol is first converted to 3-hydroxypropionaldehyde (3-HPA) by a coenzyme B_12_-dependent glycerol dehydratase (DhaB), and 3-HPA is next reduced to 1,3-PDO in a reaction catalyzed by a reduced nicotinamide adenine dinucleotide (NADH)-dependent 1,3-PDO oxidoreductase (DhaT). In addition to the reductive pathway, glycerol is metabolized by an oxidative pathway in which glycerol is dehydrogenated to dihydroxyacetone (DHA) by an NAD^+^-dependent glycerol dehydrogenase (DhaD), and DHA is next phosphorylated to dihydroxyacetone phosphate (DHAP) by an ATP-dependent DHA kinase (DhaK). During glycerol metabolism, considerable amounts of metabolites are produced via oxidative branch activity. Elimination of the production of by-product metabolites is a major strategy for engineering microbial strain for enhanced 1,3-PDO production. Among these metabolites, the major compounds are 2,3-butanediol (2,3-BDO) and lactate, attaining maximal levels of approximately 30 and 23% (w/w) that of 1,3-PDO, respectively [15].

We previously reported that fed-batch fermentation of a lactate dehydrogenase-deficient mutant of *K. pneumoniae* (Δ*ldhA*) under optimized conditions led to maximal production of 1,3-PDO from glycerol. In fed-batch fermentation, 1,3-PDO production can increase to high concentrations [15], whereas a continuous culture process can result in high productivity, but generally low concentrations, of 1,3-PDO [12]. Repeated fed-batch fermentation can compensate for the shortcomings of fed-batch and continuous culture, increasing concentration and productivity as it saved the time for sterilization, cleaning and seed culture between batches [23]. In the present study, we investigated repeated fed-batch fermentation of a *K. pneumoniae* Δ*ldhA* mutant in an effort to obtain a high production level and productivity of 1,3-PDO from glycerol. In addition, to reduce the levels of 2,3-BDO, the major by-product, we used a lactate- and 2,3-BDO-defective mutant for the repeated fed-batch fermentation.

## Results and discussion

### Effect of corn steep liquor concentration on the production of 1,3-propanediol

The cost of carbon source is the primary factor limiting the commercial production of biofuels and chemicals. Thus, much effort has been devoted to producing 1,3-PDO from inexpensive such as crude glycerol [1, 13, 14, 29]. In addition, the nitrogen source is also another key factor for the commercial production of 1,3-PDO. *Klebsiella pneumoniae* requires expensive complex nitrogen source, such as yeast extract, for 1,3-PDO production and cell growth. Corn steep liquor (CSL), a major by-product of corn-starch extraction process, could replace yeast extract as nitrogen source in culture medium [17, 19, 24]. Therefore, we investigated the effect of CSL supplementation (0, 0.5, 1.0, 2.0, 3.0, 4.0 and 5.0%) instead of yeast extract in culture medium for 1,3-PDO production and cell growth with 20 g/L initial crude glycerol in 5-L bio-reactor. Increasing the CSL concentration from 2.0 to 5.0% increased the residual crude glycerol and decreased the 1,3-PDO concentration produced after 8 h of cultivation (Table 1). Compared with the 1.0% CSL supplementation, 1 g/L yeast extract supplementation as control resulted in reaching similar levels of maximum concentration of 1,3-PDO. The maximum theoretical yield of 1,3-PDO from glycerol is 0.72 mol/mol. The production yield of 1,3-PDO reached 69.4% of the theoretical yield. Therefore, 1% CSL was chosen as the optimal supplementation for further fermentation.

**Table 1:** Production of metabolites under different CSL concentrations with 20 g/L crude glycerol

	Yeast extract (1 g/L)	CSL (%)						
		0.0	0.5	1.0	2.0	3.0	4.0	5.0
Biomass (g/L)	2.3	2.1	2.2	2.3	2.3	2.0	1.9	1.7
Consumption glycerol (g/L)	20.0	20.0	20.0	20.0	19.5	18.5	18.1	18.0
1,3-PDO (g/L)	8.3 ± 0.1	7.4 ± 0.2	7.8 ± 0.2	8.2 ± 0.2	7.7 ± 0.3	7.3 ± 0.4	7.3 ± 0.3	6.6 ± 05
2,3-BDO (g/L)	2.2	1.9	2.3	2.5	2.5	2.9	3.2	3.6
Ethanol (g/L)	1.4	1.3	1.3	1.5	1.6	1.9	1.9	2.1
Acetate (g/L)	1.7	1.5	1.9	2.0	2.1	2.1	2.1	2.2
Succinate (g/L)	0.69	0.4	0.8	0.9	0.9	0.9	0.9	0.9
1,3-PD yield (moL/moL)	0.50	0.45	0.47	0.50	0.48	0.48	0.48	0.44

### Optimization of conditions for repeated fed-batch fermentation of *K. pneumoniae* Δ*ldhA*

To establish appropriate conditions for the production of 1,3-PDO from crude glycerol with 1.0% CSL by repeated fed-batch fermentation of the Δ*ldhA* mutant strain, we first evaluated the effect of inoculum concentration (0.4, 1.6, 3.2, and 4.8 g/L) of precultured cells. Maximal levels of 100.5, 101.5, and 102.1 g/L 1,3-PDO were obtained at 60 h by cultivating precultured cells at inoculum concentrations of 0.4, 1.6, and 3.2 g/L, respectively. However, inoculation of cells at the highest concentration (4.8 g/L) resulted in a severe cell-growth defect-maximal cell growth was half that of other experiments (OD_600_ 9.2)-resulting in a low level of 1,3-PDO production. Maximal 1,3-PDO production levels and productivity at 24 h of 81.1 g/L and 3.38 g/L/h, respectively, were obtained by fed-batch fermentation with an inoculation concentration of 3.2 g/L (Table 2).

**Table 2:** Metabolite analysis of culture broths of the *K. pneumoniae* Δ*ldhA* mutant grown for 24 h by fed-batch fermentation using crude glycerol as a substrate with different inoculum concentrations

Inoculum concentration (g/L)	1,3-PD (g/L)	2,3-BD (g/L)	Ethanol (g/L)	Acetate (g/L)	Succinate (g/L)	1,3-PD (g/L/h)
0.4	63.2	27	7.2	2.1	10.3	2.63
1.6	69.9	41.4	6.6	2.0	11.2	2.91
3.2	81.1	37.5	5.7	1.5	7.9	3.38
4.8	29.2	18	6.1	1.8	6.4	1.22

Next, the effect of recycle-cell ratio in repeated fed-batch was examined. After 24 h cultivation, the culture medium was replaced with fresh medium at ratios of 1:20, 1:10, and 1:5. As shown in Table 3, maximal 1,3-PDO production levels and productivity were obtained with a fresh medium replacement ratio of 1:10.

**Table 3:** Metabolite analysis of culture broths of the *K. pneumoniae* Δ*ldhA* mutant grown for 24 h by fed-batch fermentation using crude glycerol as a substrate with different medium replacement ratios

Medium replacement ratio (%)	1,3-PD (g/L)	2,3-BD (g/L)	Ethanol (g/L)	Acetate (g/L)	Succinate (g/L)	1,3-PD (g/L/h)
5 (1:20)	68.8	30.5	6.5	2.3	8.2	2.86
10 (1:10)	79.5	39.5	6.1	1.8	11.8	3.31
20 (1:5)	78.1	40.7	7.5	1.7	12.5	3.25

### Repeated fed-batch fermentation of the *K. pneumonia* Δ*ldhA* mutant

Repeated fed-batch fermentation, was an efficient strategy to enhance the productivity of microbial products and has been applied to many fermentation strategy as it saved the time for seed culture, cleaning, inoculation and sterilization of the bioreactor between each fed-batch fermentation cycle [23]. To enhance 1,3-PDO productivity and reduce the cost of the 1,3-PDO fermentation strategy, repeated fed-batch fermentation of a *K. pneumoniae* Δ*ldhA* mutant was carried out during five cycles with 1% CSL added to fermentation medium.

**Fig. 2:**
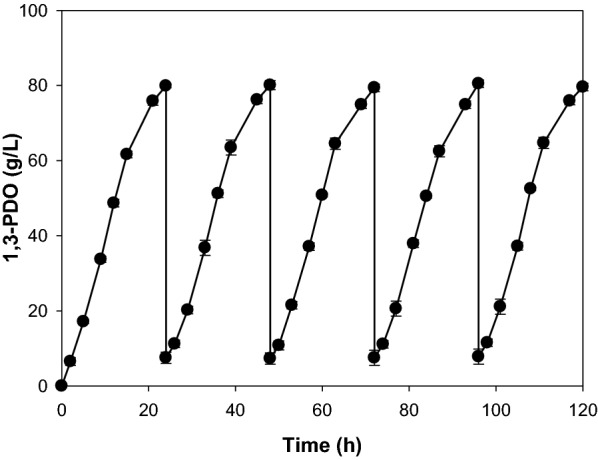
Production of 1,3-PDO by repeated fed-batch fermentation of the *K. pneumoniae* Δ*ldhA* mutant using crude glycerol as a substrate

On the bases of these preliminary results, we performed repeated fed-batch fermentation of Δ*ldhA* mutant for five cycles of 24 h each (Fig. 2). High 1,3-PDO production levels (79.9–80.5 g/L) and productivity (3.32–3.35 g/L/h) were successfully maintained during the repeated fed-batch fermentation (Table 4). Recently, Xue et al. reported a repeated fed-batch fermentation process using the *K. pneumoniae* LX3 strain that yielded a high 1,3-PDO production level (66 g/L) and high level of productivity (3.3 g/L/h) [23]. Here, optimized repeated fed-batch fermentation of the *K. pneumoniae* Δ*ldhA* mutant strain resulted in a similarly high 1,3-PDO productivity (average 3.3 g/L/h) and an even higher production level of about 80 g/L. Consistent with the lactate dehydrogenase-deficient genetic background of the mutant strain, the production of lactate, a major by-product, was completely prevented during fermentation. However, levels of the by-product 2,3-BDO were increased to up to 50% of 1,3-PDO levels.

**Table 4:** Metabolite analysis of culture broths of the *K. pneumoniae* Δ*ldhA* mutant by repeated fed-batch fermentation at intervals of 24 h using crude glycerol as a substrate

Cycle number	1,3-PDO (g/L)	2,3-BDO (g/L)	Ethanol (g/L)	Acetate (g/L)	Succinate (g/L)	1,3-PDO (g/L/h)
1	79.9	40.5	6.8	2.2	10.5	3.33
2	80.1	39.5	6.5	2.3	11.2	3.34
3	79.4	40.7	6.1	1.8	12.1	3.31
4	80.5	42.1	7.4	1.7	11.7	3.35
5	79.9	39.2	6.9	1.9	10.9	3.33

### Preparation of 2,3-BDO defective *K. pneumonia* Δ*ldhA* mutant strain

Although 2,3-BDO is a valuable chemical that is used in the production of synthetic rubber, it could severely hinder subsequent 1,3-PDO purification steps because of its similar boiling point. In order to minimize the level of 2,3-BDO production, we examined a set of mutant strains defective for each catalytic step in 2,3-BDO biosynthesis (Fig. 1): acetolactate synthase (Als), acetolactate decarboxylase (Adc), and acetoin reductase (Ard).

**Fig. 3:**
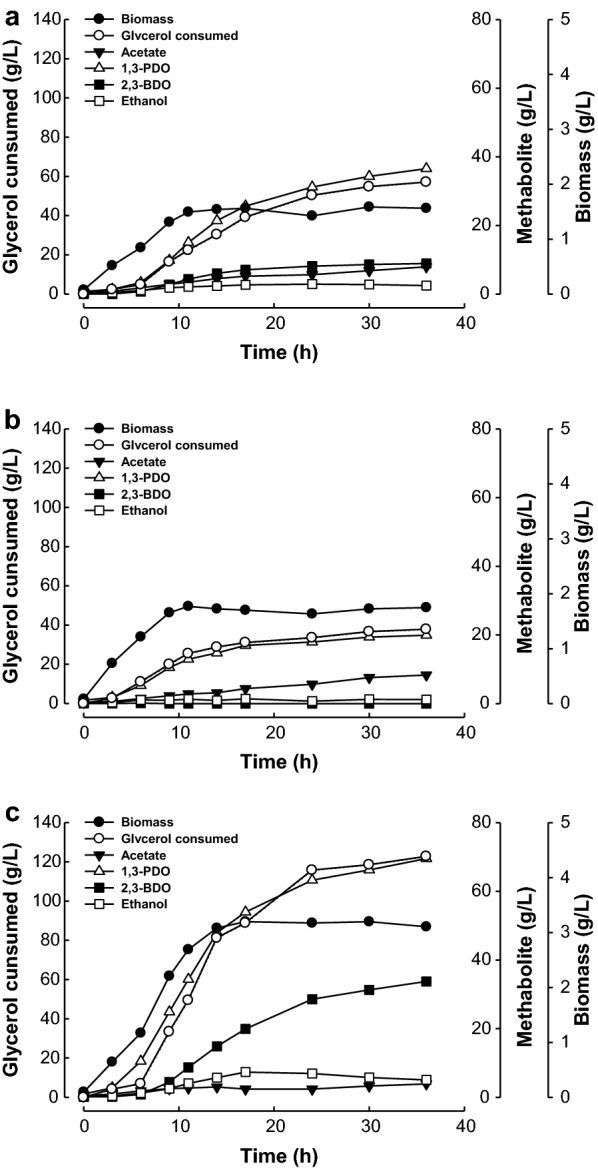
Fed-batch fermentation of *K. pneumoniae* Δ(*ldhA als*) (**a**), Δ(*ldhA adc*) (**b**), and Δ(*ldhA ard*) (**c**) using crude glycerol as a substrate

The production levels of 1,3-PDO were decreased in Δ(*ldhA als*), Δ(*ldhA adc*), and Δ(ldhA ard) mutant strains, albeit to different degrees. The most remarkable change was shown by the Δ(ldhA adc) mutant strain, in which 2,3-BDO production was completely eliminated. However, the level of 1,3-PDO production was also severely reduced in this mutant, possibly to its low growth and glycerol utilization (Fig. 3). The low growth of the Δ(ldhA adc) mutant could be caused by increased accumulation of acetate (Table 5). Although similar profiles of cell growth and metabolites production were observed in Δ(ldhA als), 1,3-PDO production was slightly higher. 1,3-PDO production was considerable in the Δ(ldhA ard) mutant strain (63.24 g/L), albeit less than that in the ΔldhA mutant (81.1 g/L), but 2,3-BDO was also co-produced at high levels. 2,3-BDO production was completely abolished in the Δ(ldhA adc) mutant, but was still observed upon fermentation of Δ(ldhA als) and Δ(ldhA ard) mutant strains, although the levels were decreased. These results indicate that isozymes of these proteins may be present in *K. pneumoniae*. In addition to the als gene, which is clustered with adc and ard genes in a region of the chromosome of *K. pneumoniae*, putative acetolactate synthase genes have been identified (KPN_00082, KPN_03085 KPN_04073, and KPN_04267). Three kinds of acetoin reductase are known in microorganisms: (2R,3R)-type, meso-type, and (2S,3S)-type [2, 8, 21]. It is known that meso- and (2S,3S)-2,3-BDO are produced by *K. pneumoniae* [8]. We found that Δ(ldhA ard) produces the (2S,3S)-type of 2,3-BDO (data not shown), indicating that the acetoin reductase encoded by ard catalyzes meso-2,3-BDO production and suggesting that another protein might be involved in the production of (2S,3S)-2,3-BDO. Unfortunately, however, homology alignment analyses failed to identify the putative gene.

**Table 5:** Metabolite analysis of culture broths of *K. pneumoniae* Δ(*ldhA als*), Δ(*ldhA adc*), and Δ(*ldhA ard*) mutant strains grown for 24 h by fed-batch fermentation using crude glycerol as a substrate

Mutant strain	1,3-PDO (g/L)	2,3-BDO (g/L)	Ethanol (g/L)	Acetate (g/L)	Succinate (g/L)
Δ(*ldhA als*)	31.20	8.14	2.91	5.66	2.79
Δ(*ldhA adc*)	17.96	0	0.73	5.62	2.02
Δ(*ldhA ard*)	63.24	28.59	6.96	2.42	7.25

Physiologically, 2,3-BDO synthesis in microorganisms is known to play a role in controlling cellular acidification by converting acidic metabolites to neutral metabolites [8, 22]. In agreement with this, the level of acetate was increased in Δ(*ldhA als*) (5.66 g/L), Δ(*ldhA adc*) (5.62 g/L), and Δ(*ldhA ard*) (2.42 g/L) mutant strains compared to that in Δ*ldhA* (1.5 g/L). The accumulation of acidic metabolites could be related to defects in cell growth, especially in Δ(*ldhA als*) and Δ(*ldhA adc*) (Fig. 3). A similar cell growth defect was also exhibited by an acetolactate synthase-deficient mutant of *K. oxytoca* [27]. We found previously that slightly acidic conditions (pH 6.0) are preferred for maximizing the production of 1,3-PDO from glycerol by Δ*ldhA* [15]. However, maximal 1,3-PDO production level and productivity by Δ(*ldhA als*) were achieved at pH 6.5 rather than pH 6.0, probably owing to a defect in the control mechanisms that regulate cellular pH. These results suggest that engineering to control acidic metabolites in 2,3-BDO-deficient mutant strains could minimize the formation of by-products and allow a further increase in the production of 1,3-PDO. On the basis of these results, we used the Δ(*ldhA als*) mutant strain for repeated fed-batch fermentation analysis.

### Repeated fed-batch fermentation of the *K. pneumonia* Δ(*ldhA als*) mutant strain

**Fig. 4:**
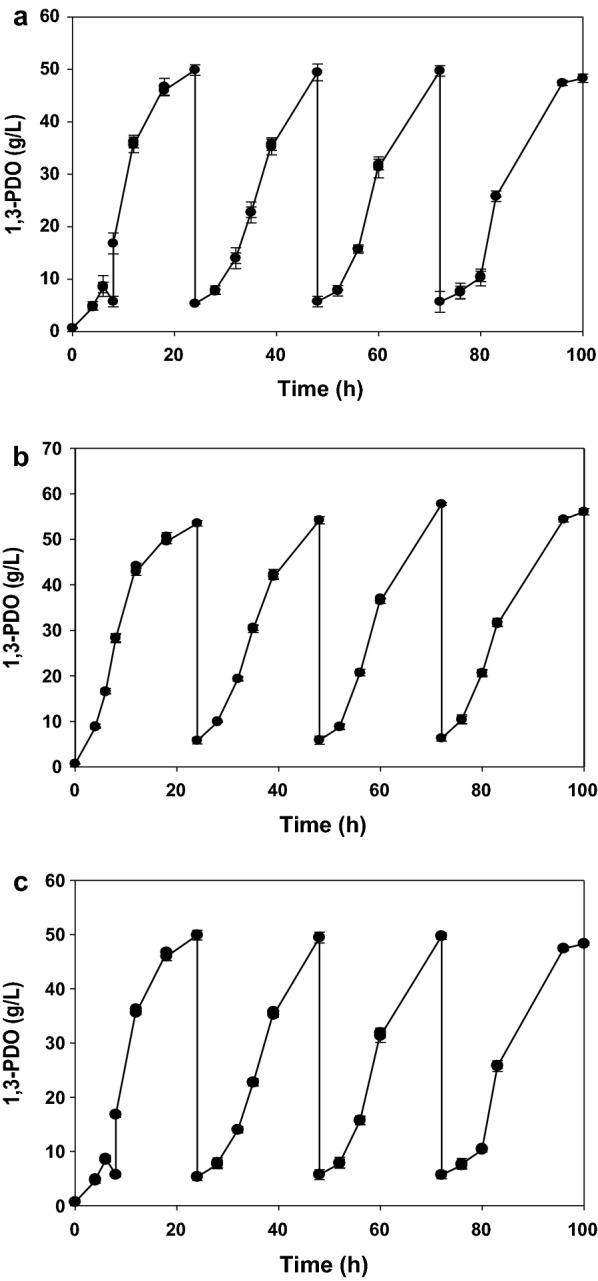
Production of 1,3-PDO by repeated fed-batch fermentation of the *K. pneumoniae* Δ(*ldhA als*) mutant at pH 6.0 (**a**), pH 6.5 (**b**), and pH 7.0 (**c**) using crude glycerol as a substrate

Repeated fed-batch fermentation of the Δ(*ldhA als*) mutant was first performed under optimized conditions (Fig. 4a). Under these conditions, the 1,3-PDO production level in the 2,3-BDO-defective mutant Δ(*ldhA als*) was 61.4% lower (average 49.1 g/L) than that in Δ*ldhA* (average 79.9 g/L). More remarkable decreases in 2,3-BDO levels were observed in Δ*ldhA* (40.4 g/L) and Δ(*ldhA als*) (9.97 g/L), resulting in 2,3-BDO:1,3-PDO ratios of 50.6 and 20.3%, respectively (Table 5).

Finally, we analyzed repeated fed-batch fermentation of Δ(*ldhA als*) under different conditions of pH (Fig. 4), a parameter that we previously showed has a major effect on the production of 1,3-PDO and 2,3-BDO in *K. pneumoniae* [13, 15]. The highest 1,3-PDO production level and productivity were observed at pH 6.5 (Table 6); these values decreased at a neutral pH.

**Table 6:** Metabolite analysis of culture broths of the *K. pneumoniae* Δ(*ldhA als*) mutant by repeated fed-batch fermentation at intervals of 24 h using crude glycerol as a substrate under different pH conditions

Cycle no.	1,3-PDO (g/L)			2,3-BDO (g/L)			Ethanol (g/L)			Acetate (g/L)			Succinate (g/L)			1,3-PDO productivity (g/L/h)		
pH	6.0	6.5	7.0	6.0	6.5	7.0	6.0	6.5	7.0	6.0	6.5	7.0	6.0	6.5	7.0	6.0	6.5	7.0
1	49.9	53.5	45.5	10.1	11.1	9.5	4.1	7.5	8.9	6.0	5.0	4.0	3.89	6.47	7.87	2.08	2.23	1.90
2	49.4	54.2	49.8	11.2	11.3	11.6	3.9	5.7	10.4	4.9	4.6	4.4	3.29	6.04	7.34	2.06	2.26	2.08
3	49.7	57.7	44.6	10.5	12.7	10.7	4.8	7.8	10.6	6.3	5.4	3.2	3.36	6.59	7.92	2.07	2.40	1.86
4	47.4	54.4	48.1	8.1	11.8	10.4	2.4	6.2	7.5	5.7	5.0	3.0	3.65	5.99	7.39	1.98	2.27	2.00

## Conclusions

This study presented experimental results of 1,3-PDO production by *K. pneumoniae* mutant under repeated fed-batch fermentation. It demonstrated that the repeated fed-batch fermentation shortened the enhanced the productivity significantly, which was of great potential for the industrial-scale 1,3-PDO production. This work offered understanding and reference for other valuable chemical production.

## Methods

### Bacterial strains, plasmids, and media

*Klebsiella pneumoniae* Δ*ldhA* and Δ(*ldhA als*), mutant strains derived from ATCC 200721 by deletion of lactate dehydrogenase gene and lactate dehydrogenase/acetolactate synthase genes, respectively, have been previously described [15]. *Escherichia coli* DH5α was used for DNA manipulations. λ Red recombinase and FLP recombinase were expressed using the helper plasmids pKD46 [5] and pCP20 [3], respectively; replication of these plasmids is temperature-sensitive, enabling the plasmids to be easily eliminated. Microbial cells were grown in LB [5 g/L yeast extract (Difco), 10 g/L Bacto-tryptone (Difco), and 10 g/L NaCl] or Germ medium [14] supplemented with appropriate antibiotics (ampicillin [50 μg/mL] and/or tetracycline [10 μg/mL for *E. coli* and 50 μg/mL for *K. pneumoniae*]). The plasmid pGEM-T Easy (Promega) was employed for cloning. Crude glycerol was obtained from a biodiesel production company (GS Bio, Yeosu, Korea). The composition of crude glycerol was glycerol 83.0% (w/w), moisture 16.5%, ash 0.2%, methanol 0.1%, MONG (matter organic non-glycerol) 0.2%.

### Construction of Δ(*ldhA adc*) and Δ(*ldhA ard*) mutant strains

The Δ(*ldhA adc*) deletion mutant was made by amplifying 300-bp DNA sequences located upstream and downstream of *adc* (KPN_02059) by polymerase chain reaction (PCR) using oligonucleotides C1 (5′-ATC GAA AAC GTC TCA AAC CAG C-3′) and C2 (5′-GAT CGT CGA GGA CGT CGG TC*G TTA AC*A TAG ACC TGA CTG CTG AAG G-3′; bases in italics indicate a *Hpa*I site) for the upstream region and C3 (5′-CCT TCA GCA GTC AGG TCT AT*G TTA AC*G ACC GAC GTC CTC GAC GAT C-3′; bases in italics indicate a *Hpa*I site) and C4 (5′-CCT TAA CTT TCT ACG GAA CGG A-3′) for the downstream region. The PCR products were annealed using the overlapping regions of the C2 and C3 primers, amplified as a single fragment using primers C1/C4, and cloned into pGEM-T Easy. The resultant plasmid was digested with *Hpa*I and ligated to an apramycin-resistance gene [*aac(3)IV*] obtained from pIJ773 by digestion with *Eco*RI and *Hin*dIII and treatment with the Klenow fragment. The resultant plasmid, designated pT-adc-Apra, was used as a template for PCR amplification of the deletion cassette, which was next introduced into *K. pneumoniae* ∆*ldhA* by electroporation [6] to induce homologous recombination. Correct integration of the DNA fragment was confirmed by Southern hybridization using the upstream regions of *adc* and *aac(3)IV* as probes of *Pst*I-digested chromosomal DNA.

**Fig. 5:**
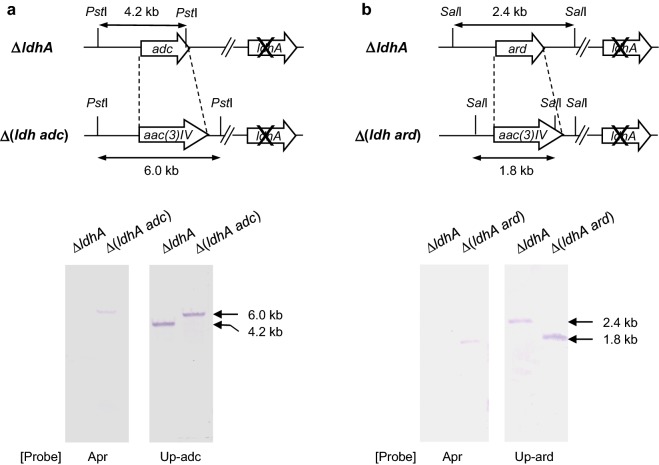
Confirmation of construction of the Δ(*ldhA adc*) (**a**) and Δ(*ldhA ard*) mutants (**b**) of *K. pneumonia* Cu by Southern hybridization. Probes: *adc*-*up* DNA sequences upstream of adc gene, *ard*-*up* DNA sequences upstream of ard gene, *Apr* apramycin resistance gene [*aac*(3)*IV*]

The method used to prepare the Δ(*ldhA*∆*ard*) deletion was similar to that described above for the construction of the Δ(*ldhA*∆*adc*) deletion. Upstream and downstream DNA sequences (300 bp in length) of *ard* (KPN_02061) were amplified by PCR using oligonucleotides R1 (5′-ATC ACA ATA AGG AAA GGA AA-3′) and R2 (5′-CGG TCA TAT AAT CAG AAT CCG *GTT AAC* CCT TTA ACG TTG ATG TTG-3′; bases in italics indicate a *Hpa*I site) for the upstream region and R3 (5′-CAA CAT CAA CGT TAA AGG *GTT AAC* CGG ATT CTG ATT ATA TGA CCG-3′; bases in italics indicate a *Hpa*I site) and R4 (5′-ATT TGG TTC CTC AAT TTT ATA G-3′) for the downstream region. The PCR products were annealed using the overlapping region of the R2 and R3 primers, amplified as a single fragment using primers R1/R4, and cloned into pGEM-T Easy. The *aac(3)IV* segment was inserted into the plasmid as described above. The resultant plasmid was designated pT-ard-Apra and was used for PCR amplification of the *adc* deletion cassette. The PCR product was introduced into *K. pneumoniae* ∆*ldhA* to induce homologous recombination. Correct integration of the DNA fragment was confirmed by Southern hybridization using the upstream regions of *ard* and *aac(3)IV* as probes of *Sal*I-digested chromosomal DNA (Fig. 5).

### Fermentation of *K. pneumoniae* mutant strains

Seed cells for fermentation were prepared in 1-L flasks containing 200 mL of defined germ medium containing 20 g/L glycerol, 2 g/L (NH_4_)_2_SO_4_, 3.4 g/L K_2_HPO_4_, 1.3 g/L KH_2_PO_4_, 0.2 g/L MgSO_4_, 0.02 g/L CaCl_2_·2H_2_O, 1 g/L yeast extract, 1 mL Fe solution [5 g/L FeSO_4_·7H_2_O and 4 mL/L HCl (37%, w/v)], and 1 mL trace element solution [70 mg/L ZnCl_2_, 100 mg/L MnCl_2_ · 4H_2_O, 60 mg/L H_3_BO_3_, 200 mg/L CoCl_2_ · 4H_2_O, 20 mg/L CuCl_2_·2H_2_O, 25 mg/L NiCl_2_·6H_2_O, 35 mg/L Na_2_MoO_4_·2H_2_O, and 4 mL/L HCl (37%, w/v)]. Flasks were incubated at 37 °C for 12 h, and cultures were subsequently inoculated into the growth vessel. Fed-batch fermentations were conducted for production of 1,3-PDO in a 5-L stirred-vessel system (Kobiotech. Co. Ltd.) containing 2-L of defined germ medium under 2.0 vvm air flow. First feeding started approximately when the residual carbon source was 10–20 g/L (about 4–8 h fermentation) in the working media. Furthermore, the crude glycerol was fed into the bioreactor to maintain its concentration between 20 and 60 g/L (i.e., the same conditions used for repeated fed-batch cultures). Repeated fed-batch culture was replaced by the fresh medium when the productivity declined, 1.8 L working medium was withdrawn through the sampling line and then same volume of fresh medium was filled into the bioreactor aseptically to start the second cycle of fed-batch culture, and the same operations were repeated five-time. All fermentation experiments were conducted at 37 °C with stirring at 200 rpm. Unless stated otherwise, the pH was maintained at pH 6.0 ± 0.2 using 28% (w/v) NH_4_OH or 2 M HCl. All data are averages from three independent experiments.

### Analytical methods

Cell growth was monitored by measuring optical density at 600 nm using spectrophotometer and then correlated with dry cell weight (DCW). For estimation of DCW, cells cultured under each condition were harvested by centrifuging at 4000×*g* for 10 min at 4 °C. Supernatants were discarded and each pellet was washed three times with phosphate-buffered saline (PBS, pH 7.2). Each pellet was transferred to a pre-weighed vial and dried at − 80 °C for 24 h using a speed vacuum concentrator (Biotron 4080C). Each vial was weighed and DCW value was estimated.

The concentrations of metabolites in culture broths were determined by high-performance liquid chromatography (Agilent System 1200) using a system equipped with a refractive index detector and an ion-exchange column (300 × 78 mm; Aminex HPX-87H; Bio-Rad). The mobile phase was 2.5 mM H_2_SO_4_ and the flow rate was 0.6 mL/min during elution. The column and cell temperatures were 65 and 45 °C, respectively [16].

## Abbreviations

*K. pneumoniae*: *Klebsiella pneumoniae*
1,3-PDO: 1,3-propanediol
2,3-BDO: 2,3-butanediol
3-HPA: 3-hydroxypropionaldehyde
ldhA: lactate dehydrogenease gene
DhaB: coenzyme B_12_-dependent glycerol dehydratase
DhaT: NAD^+^-dependent glycerol dehydrogenase
1,3-PDO: oxidoreductase
DHA: dihydroxyacetone
DhaD: NAD^+^-dependent glycerol dehydrogenase
DHAP: dihydroxyacetone phosphate
DhaK: DHA kinase
NADH: nicotinamide adenine dinucleotide
CSL: corn steep liquor
Als: acetolactate synthase
Adc: acetolactate decarboxylase
Ard: acetoin reductase
DCW: dry cell weight
